# PERSON-CENTERED PRACTICE OF NURSES IN LONG-TERM CARE HOSPITALS

**DOI:** 10.1093/geroni/igae098.3462

**Published:** 2024-12-31

**Authors:** Su Hyun Kim, Yoon Saeng Choi

**Affiliations:** Kyungpook National University, Daegu, Republic of Korea; Kyungpook National University, Daegu, Republic of Korea

## Abstract

**Objective:**

This study investigates how professional competence, professional commitment, and nursing organizational culture influence the implementation of person-centered care by nurses in long-term care facilities. Method: A sample of 131 nurses from seven long-term care hospitals in South Korea participated in this research. Standardized measures were utilized to assess professional competence, professional commitment, nursing organizational culture, and person-centered care. Data analysis was conducted using hierarchical multiple regression analysis.

**Results:**

Nurses in long-term care hospitals displayed average scores of 4.77±0.84 for professional competence and 4.27±0.89 for professional commitment on a scale of 1 to 7, and 3.45±0.44 for person-centered care on a scale of 1 to 5. Notably, nurses scored highest in relation-oriented culture and lowest in task-oriented culture. Professional competence (β=.59, p<.001) and innovation-oriented culture (β=.36, p<.001) significantly impacted person-centered care, explaining 52.5% of the variance.

**Conclusion:**

Improving person-centered care in long-term care settings requires interventions aimed at enhancing nurses’ professional competence and cultivating an innovation-oriented nursing organizational culture.

